# Development and applications of transparent conductive nanocellulose paper

**DOI:** 10.1080/14686996.2017.1364976

**Published:** 2017-08-30

**Authors:** Shaohui Li, Pooi See Lee

**Affiliations:** ^a^ School of Materials Science and Engineering, Nanyang Technological University, Singapore, Singapore

**Keywords:** Nanocellulose, transparent, electronic device, 40 Optical, magnetic and electronic device materials, 103 Composites, 201 Electronics / Semiconductor / TCOs, 209 Solar cell / Photovoltaics

## Abstract

Increasing attention has been paid to the next generation of ‘green’ electronic devices based on renewable nanocellulose, owing to its low roughness, good thermal stability and excellent optical properties. Various proof-of-concept transparent nanopaper-based electronic devices have been fabricated; these devices exhibit excellent flexibility, bendability and even foldability. In this review, we summarize the recent progress of transparent nanopaper that uses different types of nanocellulose, including pure nanocellulose paper and composite nanocellulose paper. The latest development of transparent and flexible nanopaper electronic devices are illustrated, such as electrochromic devices, touch sensors, solar cells and transistors. Finally, we discuss the advantages of transparent nanopaper compared to conventional flexible plastic substrate and the existing challenges to be tackled in order to realize this promising potential.

## Graphical abstract

**Figure:**
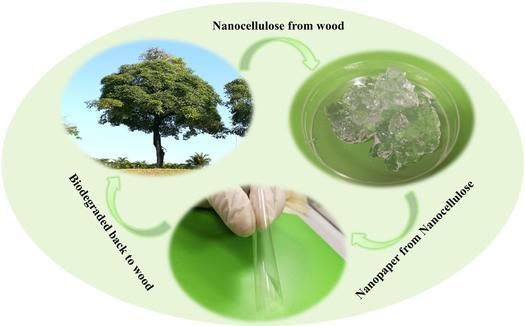

## 1. Introduction

There is growing interest in developing environmentally friendly and renewable materials, with the goal of tackling global climate change, resource shortages and waste disposal issues. As a raw material, cellulose is the most sustainable, renewable and biodegradable polymer on earth, which has been used as an engineering material for thousands of years and continues to be used today in forest products such as paper, textiles, etc. More recently, much effort has been spent on preparing flexible electronics or printable electronics on paper with the promise of excellent flexibility, low cost, light weight, inertness, recyclability and high mechanical strength when compared to the silicone-based or plastic-based electronics [1–6]. Nevertheless, the porous structure, high surface roughness, optical opaqueness or energy intensive manufacturing of the typical paper produced could not satisfy all the requirements for the next generation of ‘green’ electronics. In order to overcome these problems and to expand the opportunity of using cellulose as a host substrate for electronic devices, a thin layer of passivation is usually coated on the surface of regular papers by calendaring to reduce the surface roughness. Alternatively, the devices were first fabricated on other flat and smooth substrates like glass, and then transferred to the paper surfaces [7–10], which is more complex and incurred additional cost. However, most passivation coating alters the surface properties of the cellulose substrate and may reduce the printability of the paper due to the change of surface energy [11].

Fortunately, great understanding of cellulose fiber materials has been established in recent years. Typically, the diameter of cellulose fibers is about 20–50 μm, which is bundled of thousands of elementary microfibril with the diameter of a few or tens of nanometers [2,5,12]. By combining mechanical (homogenization, grinding, etc.), chemical (acid hydrolysis) and pre-treatment (enzymatic, oxidation) processes, the defects associated with the hierarchical structure can be removed. As a result, nanofibrils and nanocrystals of cellulose have been obtained [1,13,14]. The resultant nanocellulose paper exhibits unique optical and mechanical properties, thanks to the ease of chemical modification and reconfiguration that makes the nanocellulose an excellent candidate for the next generation ‘green’ substrate. Iwamoto et al. [15] pioneered using a grinder to treat wood pulp fiber and successfully fibrillated the fibers into nanofibrillated cellulose (NFC); further impregnation with acrylic resin led to a transparent nanocellulose-based composite paper. Saito et al. [13] made a breakthrough in preparing NFC, by using 2,2,6,6-tetramethylpiperidine-1-oxylradical (TEMPO) oxidation to pre-treat wood pulp under aqueous solution. After grinding or homogenization, the NFCs were easily disintegrated from microcellulose fibers by electrostatic repulsion from the negatively charged carboxyl groups in the C_6_ position of cellulose chains [13,16,17]. The transparency of nanopaper made from TEMPO-oxidized NFCs by vacuum filtration can reach 90% at a wavelength of 600 nm. This exciting result shows that transparent nanocellulose can compete with the optical properties of the plastic substrates. Meanwhile, bacterial nanocellulose (BNC) is also a remarkable material which has attracted considerable attention in recent years. Sharing the same structural unit as plant cellulose, BNC possesses not only the properties of conventional cellulose, but also high purity, high crystallinity and high degree of polymerization [18–21]. Usually, BNC nanopaper is not transparent due to high light scattering at the nanofibers-air interface. Yano et al. [23] first reported the use of epoxy resin impregnated into a BNC matrix to achieve a transparent BNC/epoxy nanocomposite where transmittance exceeded 80%. Over the past 10 years, research has been growing in nanocellulose, nanocellulose-based transparent conductors and nanocellulose-based devices, and several new applications have been designed and demonstrated, such as electrochromic and energy harvesting devices, touch panels, etc.

The goal of this review is to provide an overview of recent developments and challenges in developing transparent nanocellulose paper, including the material preparation process and device applications using transparent nanocellulose paper.

## 2. The structure and types of nanocellulose

**Figure 1.**
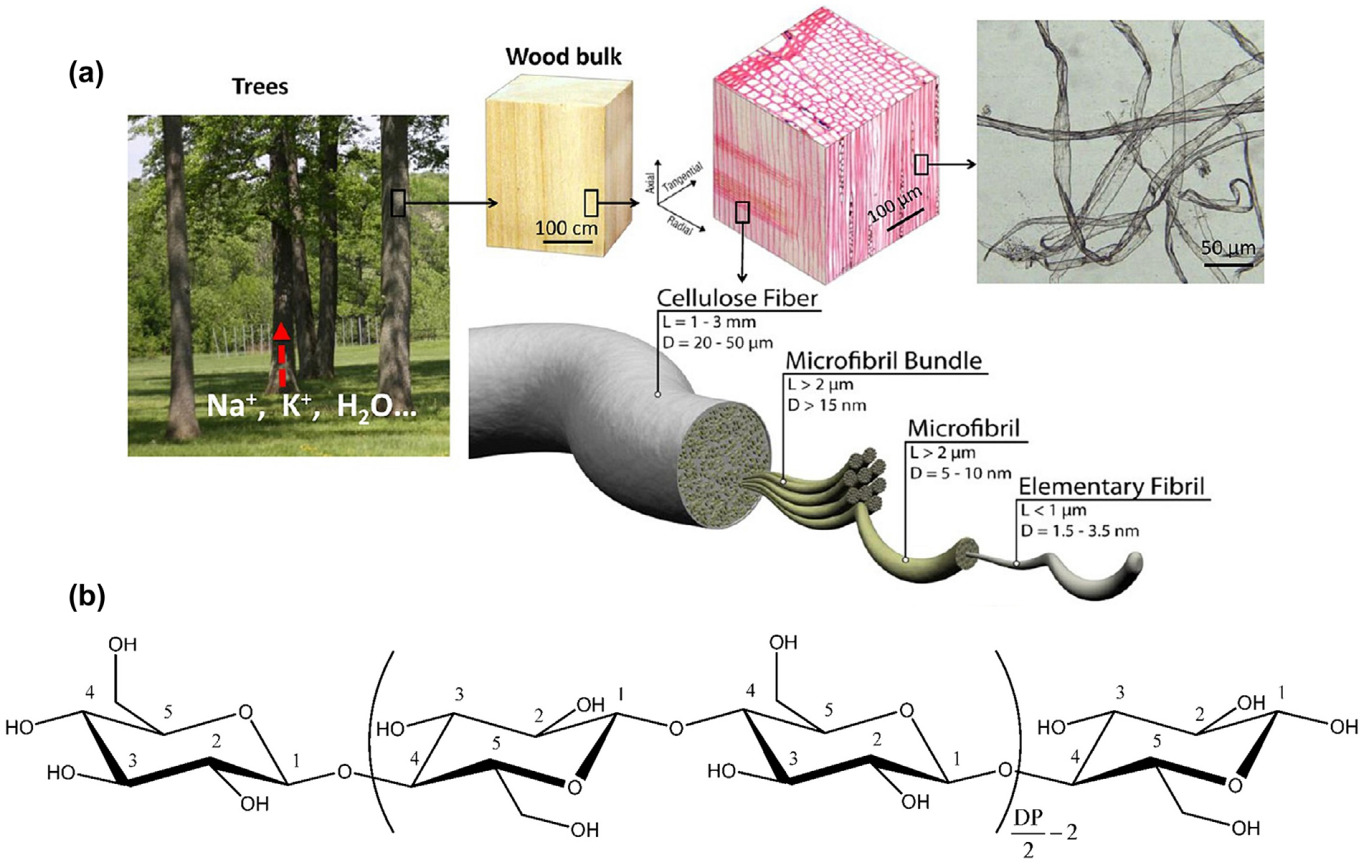
(a) Schematic of hierarchical structure of wood fibers [2]. Reproduced with the permission of [2]. Copyright 2013 The Royal Society of Chemistry. (b) Chemical structure of cellulose [12]. Reproduced with the permission of [12]. Copyright 2010 American Chemical Society.

Cellulose is the most environmentally friendly, renewable and abundant biopolymer on earth, which is the major component of lignocellulosic plant biomass. Currently, most cellulose comes from wood, in which 40–45% fibers are composed of cellulose. Usually, cellulose fibers have a length of about 1–3 mm and a diameter of 20–50 μm. They consist of bundles with a length greater than 2 μm and a diameter wider than 15 nm. Individual fibrils in those bundles have the same length as the bundle, but a narrower diameter of about 5–10 nm. These fibrils are built from elementary units with a length <1 μm and a diameter ~1.5–3.5 nm. This complex hierarchical structure of wood fibers is shown in Figure 1(a) [24]. Cellulose can be characterized as a biopolymer which is assembled from β-1,4-linked anhydro-D-glucose units. Every unit is corkscrewed 180º relative to its neighbors, as shown in Figure 1(b) [5,12]. Using different methods, two types of nanocellulose (nanocrystalline cellulose and nanofibrillated cellulose) can be isolated from plant cellulose. A third type of nanocellulose, BNC, is produced by bacteria. In this section, we focus on the preparation and properties of nanocellulose.

### 2.1. Nanocrystal cellulose (NCC)

NCC contains rigid rod-like particles resulting from the chemical treatment of cellulose. NCC was first reported by Ränby et al. [25] in 1950. They obtained a colloidal suspension of cellulose after the sulfuric acid hydrolysis of cellulose fibers. This material was then studied by Nickerson and Habrle [26] and extensively investigated in 1960. The main method to isolate NCC from cellulose fibers is based on chemical treatment in a mild acid hydrolysis. The acid can be easily infiltrated into the disorder or amorphous regions of cellulose and causes it to be hydrolyzed, whereas the crystalline regions can be maintained due to their higher resistance to acid attack. Thus, after acid treatment, rod-like nanocrystals are produced. The morphology and crystallinity are similar to those of the original. Various acids are studied to isolate NCC, such as hydrochloric [27], phosphoric [28], and hydrobromic acid [29]. Sulfuric and hydrochloric acid were extensively used to isolate NCC, especially sulfuric acid. Sulfuric acid can react with the surface hydroxyl group, introducing negatively charged sulfate groups on the NCC surface [30]. These surface groups induce repulsive forces and promote the dispersion of NCC in water. Many researchers have studied the isolation conditions and the effect of the raw material for its yield, dimensions and properties [12,30].

**Figure 2.**
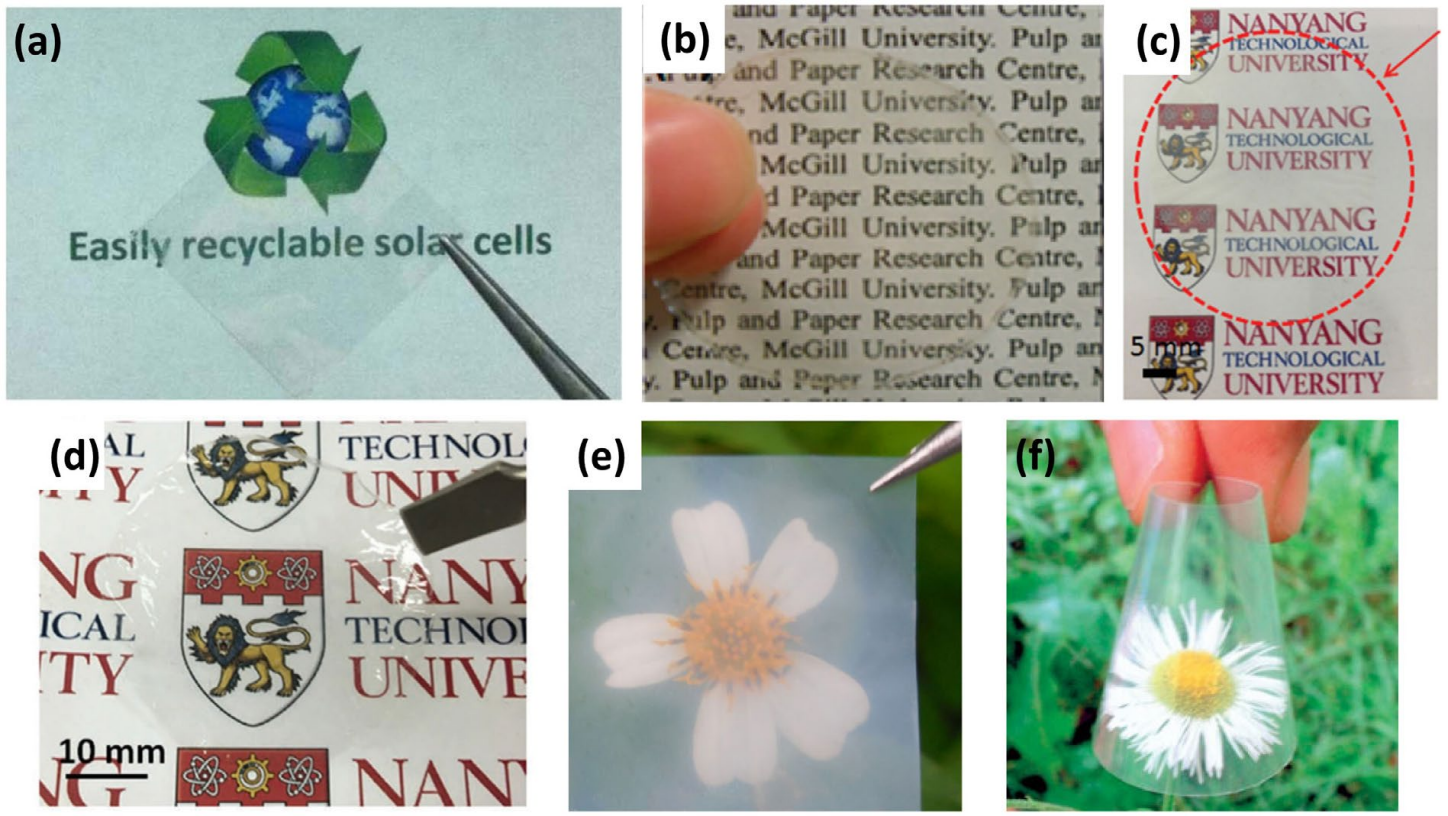
Various transparent papers made of different cellulose materials. (a) Transparent nanocomposite paper prepared by using NCCs and glycerol [31]. Reproduced with the permission of [31]. Copyright 2013 Nature Publishing. (b) Transparent nanopaper prepared by using NCCs [32]. Reproduced with the permission of [32]. Copyright 2012 American Chemical Society. (c) Transparent nanopaper obtained from NFCs by vacuum filtration [41]. Reproduced with the permission of [41]. Copyright 2015 Wiley-VCH. (d) Transparent nanopaper made of TEMPO-oxidized NFCs by vacuum filtration [42]. Reproduced with the permission of [42]. Copyright 2016 Wiley-VCH. (e) Transparent nanopaper made of BC sheet [22]. Reproduced with the permission of [22]. Copyright 2016 Springer Publishing. (f) Transparent nanocomposite paper prepared by using BC sheet with acrylic resin [23]. Reproduced with the permission of [23]. Copyright 2005 Wiley-VCH.

The dimensions of NCCs vary from 10 to 50 nm in width and 100 to 500 nm in length depending on the starting materials and hydrolysis conditions [30]. Due to their high stiffness, pure NCC films are very fragile, which limits their application in flexible electronics. Consequently, only a few papers have reported on the use of NCC film in electronic devices. Zhou et al. [31] have prepared a NCC and glycerol composite nanopaper by casting (Figure 2(a)), where the nanopaper exhibited very low roughness (root-mean-square value of 1.8 nm). The organic solar cell fabricated on the nanopaper surface can reach a power conversion efficiency of 2.7%. Yang et al. [32] developed a method to produce electrostatically stabilized NCC. Nanopaper prepared using this modified NCC shows a high transparency up to 87% (Figure 2(b)).

### 2.2. Nanofibrillated cellulose (NFC)

After Turbak et al. [33,34] first extracted NFC from wood fibers by mechanical processes in 1984, there has been growing interest in making transparent nanopaper from NFC over the past decades. Taniguchi and Okamura et al. [35] first reported film preparation by using natural microfibrillated fibers with a diameter of 20–90 nm. Usually, the structure of NFC is composed of both the amorphous and crystalline parts of the original cellulose fiber, which can be isolated from cotton, wood and annual plants by mechanical treatment. The fibers possess a high aspect ratio (~100) with a length of a few micrometers and width of 2–60 nm [3]. However, due to the strong hydrogen bonding between the nanofibers, the mechanical fibrillation requires significant time and energy, which is a big barrier for commercial applications. In order to reduce the time and energy consumption during the mechanical disintegration, various pre-treatments such as chemical [17,36], alkaline [37,38] and enzymatic treatments [39,40] have been developed in recent years. Among these pre-treatments, TEMPO oxidation under aqueous conditions is a low cost and effective method which can facilitate disintegration from microscopic wood fibers and reduce the number of passes over apparatus. Transparent nanopapers made from TEMPO-oxidized NFCs possessed excellent transparency and high barrier for gas. The nanopaper prepared by using NFCs and TEMPO-oxidized NFCs are shown in Figure 2(c) and (d), respectively [41,42]. The transparent nanopaper reported by Fukuzumi et al. [43] from a softwood TEMPO-oxidized NFC exhibits high optical transparency (up to 90% at the 600 nm wavelength) and a low coefficient of thermal expansion (~2.7 ppm K^−1^ m). The reduction of time and energy consumption helped to realize the industrialization of NFC after 2010 [44]. Currently, most reported transparent nanopaper is prepared by pre-treatment with TEMPO and then mechanical fibrillation by grinding, microfluidization or homogenization, to obtain gel-like NFCs in water at low concentrations (1–2%) [2,41]. To commercialize transparent nanopaper made of NFC or NFC-based composites, scientists have attempted to develop efficient manufacturing techniques. Sehaqui et al. [45] prepared a nanopaper by using a semiautomatic sheet former, whereby nanopaper with a thickness of 60 μm can be completed in one hour, but the transparency of the obtained nanopaper is only 42% at 600 nm wavelength. Varanasi and Batchelor [46] reported a rapid method which can prepare nanopaper in 10 minutes. Recently, Xu et al. [47] reported a low-haze hybrid transparent paper comprising long NFCs and short NCCs. By controlling the NFC/NCC ratio in the paper, the transmittance and haze can be tailored to accommodate different applications. To improve the yield of NFCs, Chen et al. [48] reported a partial dissolution method, whereby the yield can reach 56.5% and sodium carboxymethylcellulose can be produced at the same time. Although significant progress in the fabrication high transparent nanopaper has been developed in the last few years, there is still a lack of an efficient method to manufacture nanopaper on a large scale, which limits its widespread application in the flexible electronics area.

### 2.3. Bacterial nanocellulose (BNC)

Different from the rod-like NCCs and fiber-shape NFCs isolated from plant cellulose, BNC is produced by *Acetobacter xylinum* and some other species, which consists of ribbon-shape nanofibers in a web-like network [20,21,23]. BNC shares the same structural unit as NFC and NCC, which possess some specific properties, such as high crystallinity, high chemical purity (about 100%), high degree of polymerization and ultrafine web-like structure; a typical BNC film is shown in Figure 2(e) [22]. These properties make re-dispersion of BNC difficult. Even after treatment with homogenizer, the nanofibers are still bound to each other by the abundant hydrogen bonds. Yano et al. [23] first reported a transparent composite nanopaper by impregnating epoxy resin into the BNC. The fiber content in BNC/epoxy composite is as high as 70% (Figure 2(f)), which also exhibits a low thermal-expansion coefficient and high mechanical strength. However, the hygroscopicity of the cellulose causes it to be dimensionally unstable, which can be detrimental for use in optoelectronics. In order to solve this problem, they reinforced the BNC nanofibers with acetylate, which can efficiently reduce moisture absorption without sacrificing optical transparency and thermal stability [49]. They have succeeded in depositing an electroluminescent layer on this transparent BNC nanocomposites. Nogi et al. [50] reported a flexible plastic BNC nanocomposite by impregnating BNC with low-Young’s-modulus transparent resin; this reinforced nanopaper exhibits excellent flexibility and can be folded or bent without damage. Wu et al. [22] oxidized the BNC nanofibers suspension with TEMPO, and then prepared the film by a cast-drying method. The film shows high transparency with 83% transmittance at a wavelength of 550 nm, high Young’s modulus of 9 GPa, high tensile strength of 163 MPa and low coefficient thermal expansion of 3.2 ppm K^−1^. Although the transparent BNC nanopaper can be prepared by using impregnated resin or TEMPO pre-treatment with excellent transparency, more improvements are still required before it can be compared with the NFC nanopaper.

Biodegradability is an important advantage of nanopaper. Jung et al. [51] studied the fungal biodegradation of pure NFC film and epoxy-coated NFC film. After 28 days, the average weight loss of pure NFC was 35.2%, while the average loss of the epoxy-coated NFC sample was 6.6% due to the effect of epoxy. The degradation of a memory device using NFC nanopaper as a substrate was studied in natural soil by Celano et al. [52]. After removal of the Ag electrode from the device, the degradation of the nanopaper started after 15 days, and fully degraded after 26 days. These results show that nanopaper can be degraded in nature, but for electronic devices, they should be pre-treated to remove the nondegradable part before being decomposed in nature, such as the Ag electrode or the epoxy protective layer.

## 3. Applications of transparent conductive nanocellulose paper

Electronics, such as mobile phones, computers, TVs, solar cells, sensors and cameras, play an important role in our daily lives. These electronic devices are replaced frequently and discarded with a short upgrading period, which poses a growing environmental problem due to the wide use of plastics, glasses and silicones as substrates, which are non-biodegradable and non-recyclable. Transparent nanocellulose paper electronics attract broad attention in academic research and industrial applications for their environmentally friendly nature, sustainability, cost-efficient preparation, light weight, excellent optical properties and capability with large-scale roll-to-roll processes. A variety of transparent paper-based electronic devices have been designed and fabricated over the past 10 years. In this review, we mainly summarize the recent progress in electrochromics, touch sensors, solar cells, transistors and organic light-emitting diodes (OLEDs).

### 3.1. Electrochromic devices

**Figure 3.**
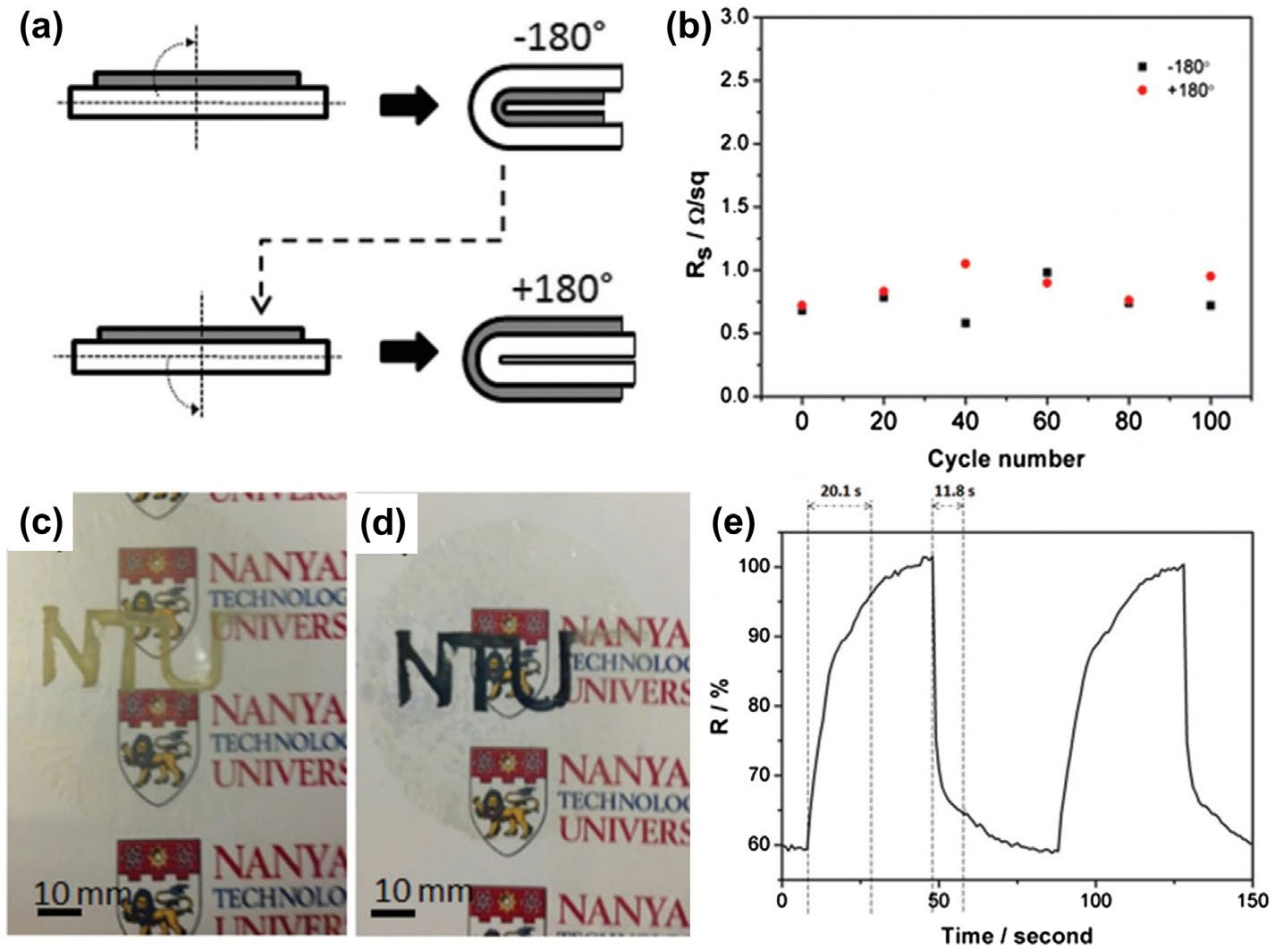
Schematics for the folding procedure. (b) Sheet resistance versus the number of ±180° folding cycles. Images of the EC nanopaper in the (c) bleached state and (d) colored state. (e) Switching rate test [41]. Reproduced with the permission of [41]. Copyright 2015 Wiley-VCH.

**Figure 4.**
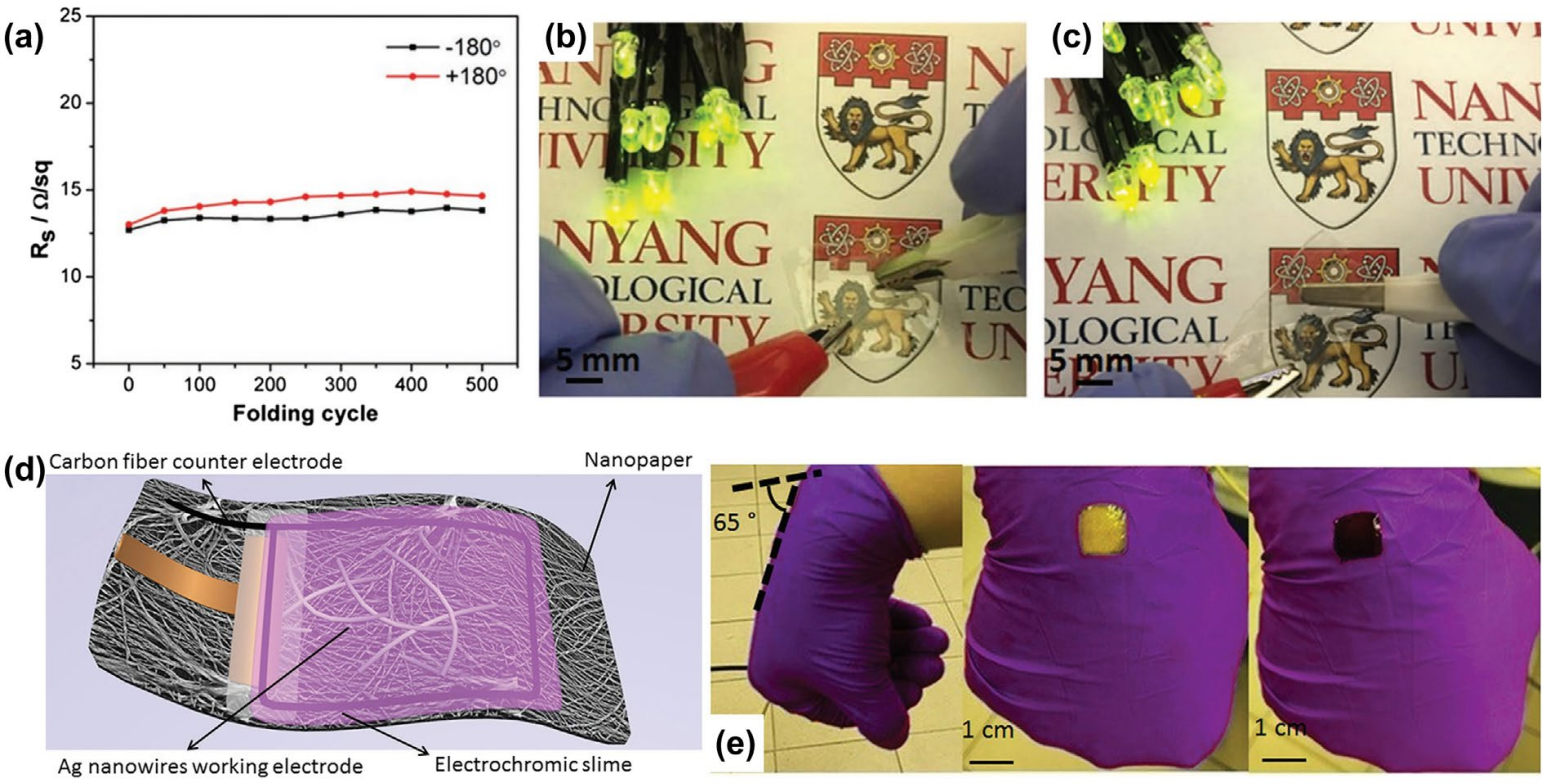
(a) Sheet resistance versus the number of ±180° folding cycles. Images of the foldability test of the SWCNT@Ag nanopaper electrode (b) before and (c) after crumpling. (d) Schematic of the solid state EC device. (e) Demonstration of solid state EC device mounted on a bent wrist [42]. Reproduced with the permission of [42]. Copyright 2016 Wiley-VCH.

Electrochromic (EC) devices enable reverse changes to their optical properties under the control of an electric field, which shows potential applications in smart windows, information displays, antiglare mirrors and EC e-skins. Usually, EC devices use glass and polyethylene terephthalate (PET) as substrates with coated tin-doped indium oxide (ITO) or fluorine-doped tin oxide (FTO) as the transparent conductors, which limit their applications in flexible devices due to the brittleness of ITO and FTO. Much effort has been channeled into using carbon nanotubes (CNTs) [53,54], metallic wire networks [55,56], graphene [57,58] and metal grids to prepare the transparent conductor [59,60]. Flexible transparent conductors have been significantly improved in recent years by combining them with plastic substrates, such as polyethylene terephthalate (PET), polyimide (PI) and polycarbonate (PC), However, the plastic materials are non-recyclable and unsustainable environmentally. Increasing attention has been paid to the versatile, sustainable and environmentally friendly nanocellulose paper-based transparent conductors. Kang et al. [41] prepared a nanocellulose-Ag nanowire (AgNW) transparent conductor by utilizing a transfer method. The transmittance of the transparent conductor can reach 84.5% with a sheet resistance of 59.7 Ω sq^−1^. The nanopaper-based conductor shows excellent foldability. Figure 3(a) and (b) show the effect of repeated ±180º folding. The sheet resistance of the electrode only shows slight increase from 0.68 Ω sq^−1^ to 1 Ω sq^−1^. To demonstrate the application of the transparent conductor in EC, a patterned EC electrode is prepared by electrodeposition of a WO_3_ layer (Figure 3(c) and (d)). As shown in Figure 3(e), a fast coloring and bleaching time is calculated to be 11.8 and 20.1 s, respectively. The nanopaper can be quite effective in relieving the intercalation stress, leading to improved electrochemical stability. After 500 cycles of stability test of the EC electrode, the contrast lost is only 22%. The excellent EC performance makes it promising as the next-generation of flexible e-papers or displays. Later, Kang et al. [42] prepared a kinked AgNW and a single-wall CNTs (SWCNTs)@AgNW transparent conductors on nanocellulose paper through the same transfer method. As the kinked structure can effectively reduce the junction resistance, both types of conductors exhibit excellent conductivity and high transparency. Additionally, the SWCNTs not only help to bridge and fuse the junction of AgNWs to improve conductivity, but also help to anchor the AgNWs and protect them from external deformation, which can improve the foldability of the transparent conductors. After 500 folding cycles with 6 μm bending radius, the sheet resistance only increased slightly with 8.8% and 12.7% for −180° and +180° folding, respectively (Figure 4(a)). Even after crumpling, the nanopaper-based transparent conductor retained its conductivity (Figure 4(b) and (c)). To demonstrate potential applications for the nanopaper transparent conductor, a solid state foldable EC is fabricated by using a transparent nanopaper conductor as a working electrode, carbon fiber as a counter electrode and solid state electrochromic slime as active materials. The structure of the full device is shown in Figure 4(d). The solid state electrochromic device can color and bleach at the potential of −2 V and 0 V, respectively. The switching times t_coloring_ and t_bleaching_ are about 11.5 and 12.9 s, respectively. The device also exhibits excellent foldability and cycling stability (Figure 4(e)). After 100 folding cycles, the switching time of t_coloring_ and t_bleaching_ increased slightly to 13.6 and 17.9 s, respectively. The contrast retention of 73.3% can be achieved after 500 cycles. These results show the potential applications of a nanopaper transparent conductor in EC for next-generation deformable displays such as foldable information displays and camouflage clothing.

### 3.2. Touch sensors

**Figure 5.**
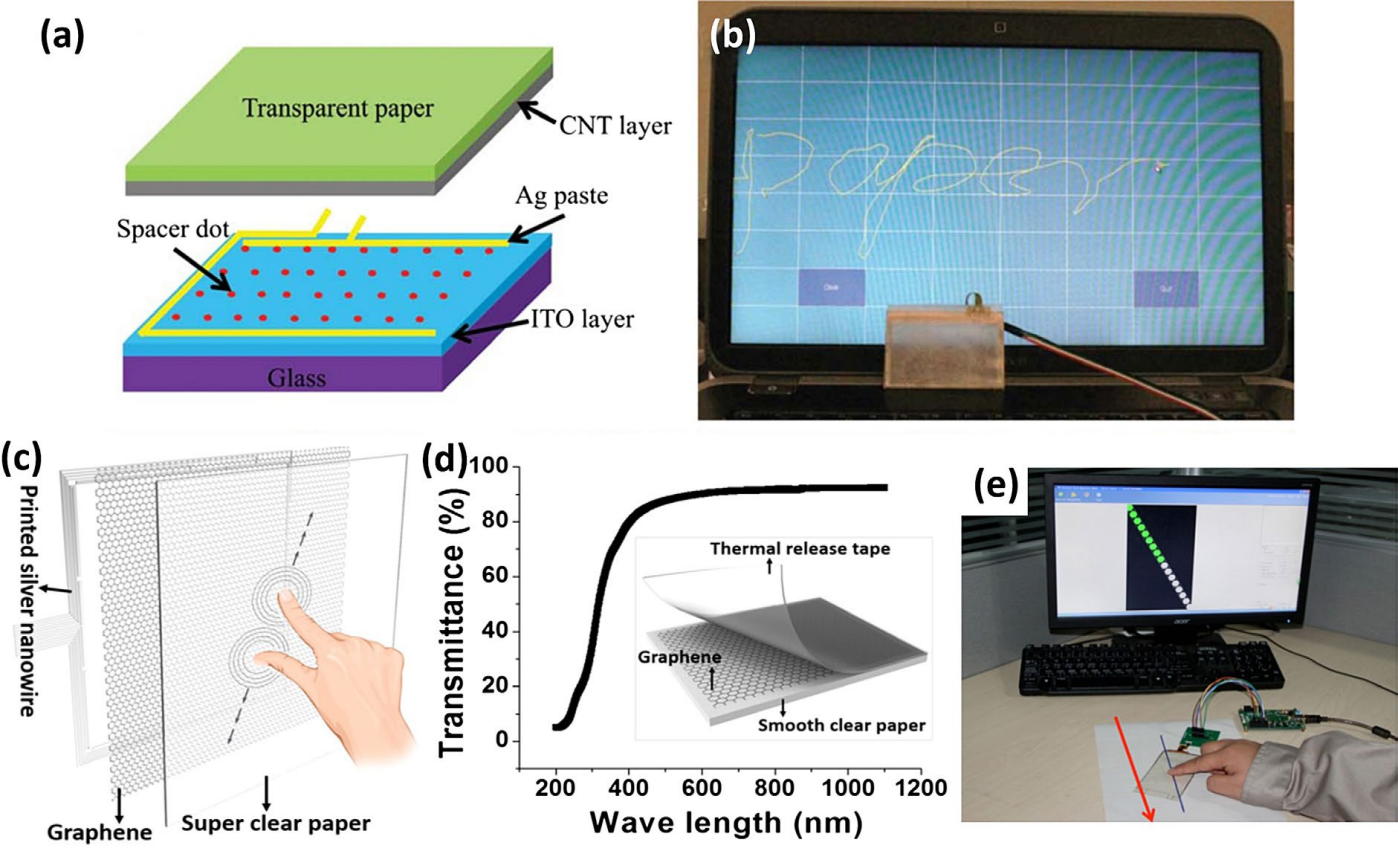
(a) Schematic structure of a four-wire resistive touch screen with CNT and transparent nanopaper electrode. (b) The word ‘paper’ was demonstrated in an assembled paper touch screen [4]. Reproduced with the permission of [4]. Copyright 2013 The Royal Society of Chemistry. (c) Schematic of super clear paper-based multipoint capacitive touch screen. (d) Optical of the whole touch screen device. (e) Measurement of linearity of paper-based touch screen [62]. Reproduced with the permission of [62]. Copyright 2016 American Chemical Society.

Paper-based touch sensors have attracted tremendous attention since they are lightweight, portable and flexible. Fang et al. [4] designed a bilayer transparent nanopaper by using regular wood fibers as the backbone and NFC as fillers. Such a hybrid nanopaper exhibits excellent optical transmittance and superior surface roughness. A thin layer of CNT was then deposited on the surface by rod coating to make it conductive. A four-wire resistive touch screen was fabricated using this transparent conductive paper. The structure of the device is shown in Figure 5(a). This sensor can sense physical touch, with the signal being transferred to the computer with an external controller. A letter ‘paper’ was written on the touch sensor by a stylus pen and successfully displayed on the computer (Figure 5(b)). This is the first reported transparent paper as a substrate for touch screen applications. Ji et al. [61] reported a transparent cellulose nanofiber (CNF) hybrid film by using an electrospinning polymer as the backbone and sprayed CNF as fillers. The resulting hybrid film presents outstanding optical transparency and a low coefficient of thermal expansion. After spin-coating with AgNWs, a four-wire resistive touch screen panel was fabricated by utilizing the hybrid films as transparent electrodes for the touch panel. This transparent and flexible touch panel also shows excellent sensory performance. Furthermore, the thermal stability of the integrated touch panel is also very good. After exposure to a temperature of 100 °C for 10 days, the hybrid film was stable with no cracking observed on the gold interconnections. Zhu et al. [62] prepared a highly transparent and clear nanopaper with transmittance >90% and haze <1.0%. To make it conductive, a single layer graphene synthesis by chemical vapor deposit (CVD) was dry-transferred onto the nanopaper as a transparent conductive electrode. The sheet resistance is about 445 Ohm/sq and the transmittance is 89% in the visible range. In order to show the practical applications of this paper, the electrode was patterned with a laser cutter. A multi-touch capacitive touch screen based on the nanopaper substrate could be fabricated using the same process as for commercial plastic-based touch screens. Figure 5(c) illustrates the structure of the touch screen and Figure 5(d) shows the device exhibits as highly transparent. The sensory performance of the super clear paper touch screen is comparable to the commercial touch screen (Figure 5(e)).

### 3.3. Solar cells

**Figure 6.**
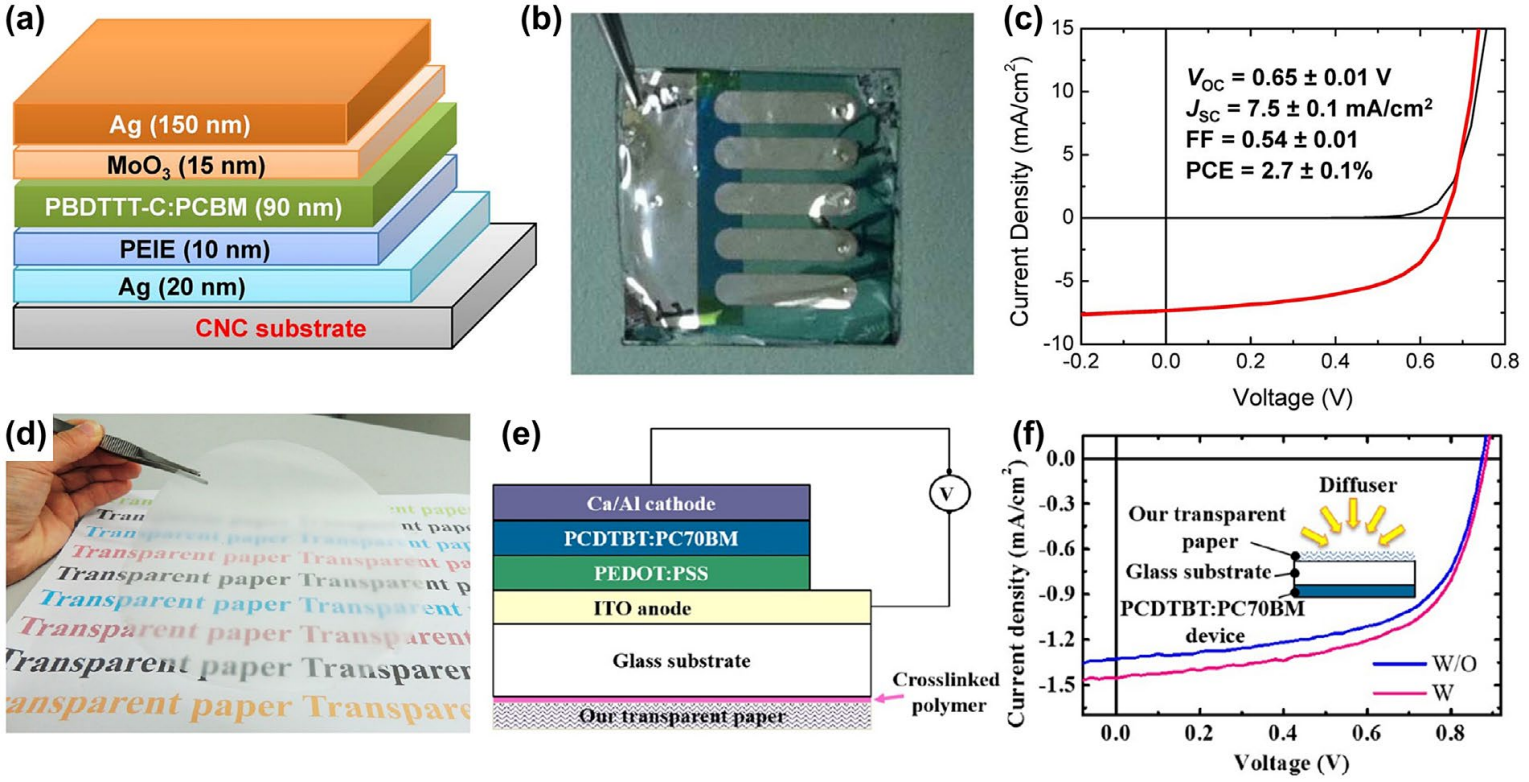
(a) Schematic structure of the organic solar cell on NCC substrates. (b) Image of an assembled solar cell. (c) I-V curve of the solar cell on NCC substrates [31]. Reproduced with the permission of [31]. Copyright 2013 Nature Publishing. (d) Digital image of the transparent nanopaper with ultra-high haze prepared from TEMPO-oxidized wood fibers. (e) Structure of the solar cell device with ultra-high haze nanopaper attached on the opposite glass side. (f) I-V curves of the solar cell with/without transparent nanopaper made of wood fibers [65]. Reproduced with the permission of [65]. Copyright 2014 American Chemical Society.

A benefit of the high optical haze of transparent nanopaper is that it is very attractive for high-efficiency organic solar cells with light management. Significant advances have been made in the past several years. Zhou et al. [31] demonstrated an efficient organic solar cell by using optical transparent NCC film as a substrate: the structure of the device is shown in Figure 6(a). The assembled device is displayed in Figure 6(b). The solar cell displays good rectification in the dark. A power conversion efficiency (PCE) of 2.7% can be obtained (Figure 6(c)). The values of circuit voltage and fill factor are similar to the solar cells by using ITO/glass. Though the PCE is lower than the ITO/glass-based solar cell (PCE of 6.6%), this device exhibits attractive technology for sustainable and environmental energy production. The solar cells can be easily separated and recycled using low energy at room temperature. Hu et al. [63] reported a highly transparent with large light scattering nanopaper, which can be successfully deposited with conductive materials, like ITO, carbon nanotubes and silver nanowires. This transparent conductive paper can be used in many applications such as displays, touch screens and solar cells. They demonstrated an organic bulk of heterojunction solar cells, and a PCE of 0.4%. The cell efficiency can be further improved by controlling surface smoothness during fabrication. Nogi et al. [64] prepared a transparent conductive nanofiber paper by using AgNWs and NFCs. Due to the hydrophilic affinity between cellulose and AgNWs, the hybrid paper exhibits good foldability, without a change in the resistance after 20 times folding cycles. The organic solar cell was fabricated using this transparent conductive paper as a substrate. Its PCE can reach 3.2%, which can be compared with the ITO base solar cells. Usually, the transparent nanopaper film exhibits a transparency of about 90% and low optical haze (<20%), properties which can be modulated with different structures. Fang et al. [65] prepared a novel low-cost transparent paper with ultra-high optical transparency (~96%) and ultra-high haze (~60%) (Figure 6(d)). This is a perfect substrate for solar cells. The majority of the transmitted light was scattered off in a normal direction, which can increase the optical path length in the active materials and improve optical adsorption. By directly attaching this transparent paper to the glass side of the organic photovoltaic device, the PCE was increased from 5.34 to 5.88% due to the increased photocurrent (Figure 6(e) and (f)). Ha et al [66]. used a transparent paper-based anti-reflection coating layer to improve the efficiency of the solar cell. The paper showed angle independent behavior at all wavelengths, which can significantly decrease the light reflectivity and improve the cell’s PCE. After attaching a transparent paper on the GaAs solar cell, the PCE was increased from 13.55% to 16.79%.

### 3.4. Transistors

**Figure 7.**
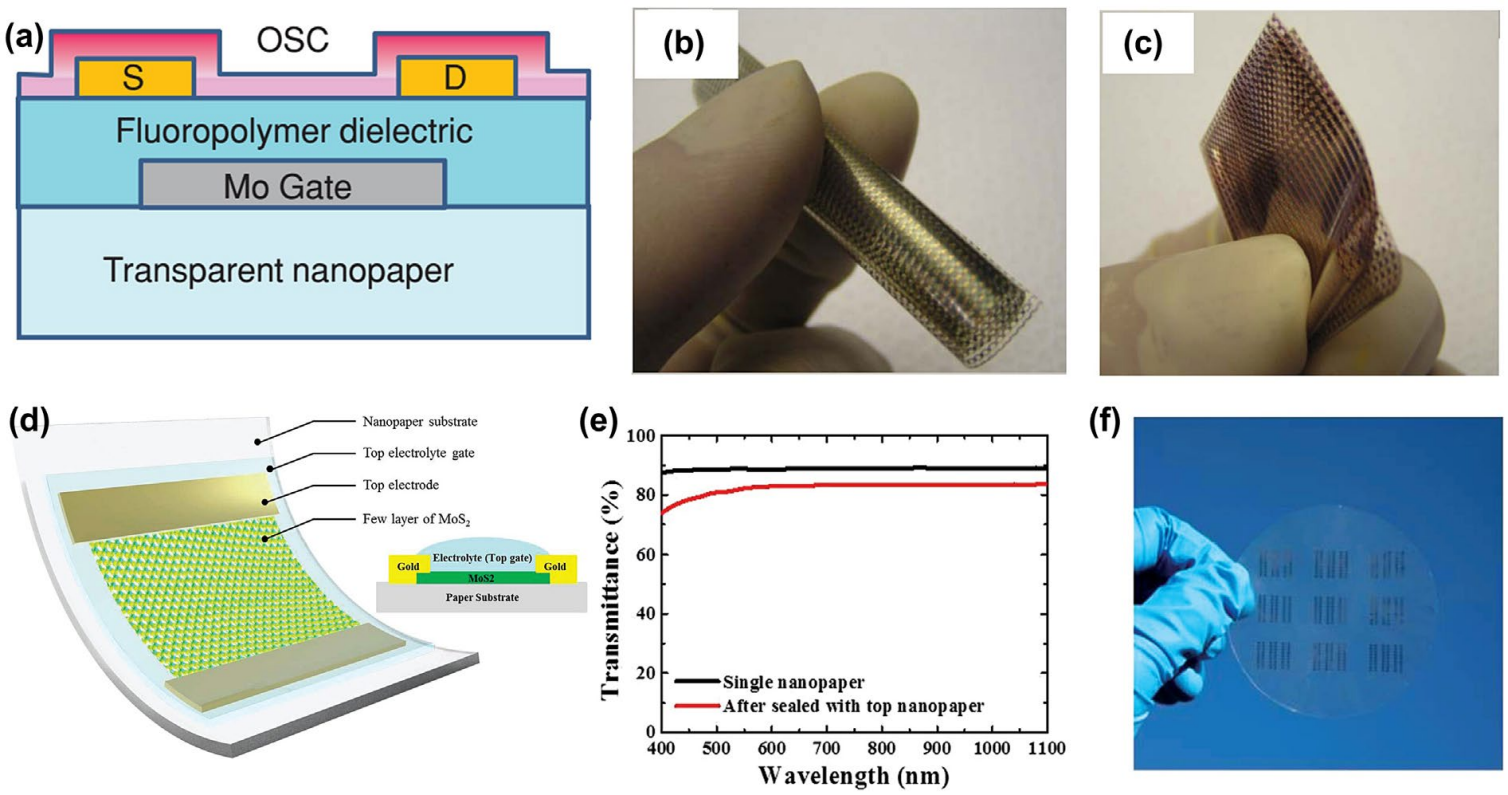
(a) Cross section of fabricated organic TFT on nanopaper substrate. Photographs of 20-μm-thick transparent nanopaper-based organic TFT array in (b) bending and (c) folding states [68]. Reproduced with the permission of [68]. Copyright 2014 Wiley-VCH. (d) Schematic and cross section of MoS_2_ phototransistor with flexible nanopaper as substrate. (e) Optical transmittance of nanopaper and the sealed phototransistor. (f) Image of an array of phototransistor [69]. Reproduced with the permission of [69]. Copyright 2016 The Royal Society of Chemistry.

Thin film transistor (TFT) is one important component for microelectronic devices to amplify or switch electronic signals. Different from regular paper, nanocellulose paper exhibits low surface roughness, strong mechanical strength and excellent optical properties, which make it very suitable as a flexible and transparent substrate to fabricate TFT. Huang et al. [67] reported a highly transparent and flexible organic transistor device by using a NFC paper as substrate. The nanopaper transistor exhibits good electrical characteristics; the carrier mobility is around 4.3 × 10^−3^ cm^2^/(Vs) and I_on_/I_off_ ratio can reach up to 200. Flexibility tests indicated that the transfer curves exhibited no significant change upon blending in different directions. The mobility only decreased 10% under bent conditions, showing great potential in the next generation of transparent and flexible electronics. Fujisaki et al. [68] fabricated a short-channel bottom-contact organic TFT array on the NFC substrate; the structure is shown in Figure 7(a). The dielectric layer was spin-coated on the nanopaper, which exhibits a low surface roughness and low current leakage (below 10^−8^ A cm^−2^). Owing to the smoothness of the dielectric layer, the top organic semiconducting layer was also spin-coated. The TFT device exhibits a high on-current of 10^−5^A, high on/off ratio of 10^6^–10^8^ and a mobility of 1 cm^2^ V^−1^s^−1^ under ambient air. This TFT performance was comparable with the results on a plastic substrate. Moreover, the thin nanopaper-based TFT array also exhibits excellent flexibility and mechanical durability, which can be bent and folded without tearing or fracturing. The mobility shows no degradation after operation under 1H of bending (see Figure 7(b) and (c)). Zhang et al. [69] successfully prepared a MoS_2_ phototransistor on cellulose nanopaper; the corresponding schematic and cross-sectional structure is shown in Figure 7(d). This device exhibits an exceptional photoresponsivity of ~1.5 kAW^−1^ and excellent optical transmittance of 82% (Figure 7(e)), which suggests the potential for various applications such as touch panels, solar cells and image sensors. An array of phototransistor is displayed in Figure 7(f), showing highly transparency. The above reports focus on transparent nanopaper as substrate; there are also some reports using nanopaper as both substrate and dielectric layers. Gaspar et al. [70] demonstrated a thin transparent nanopaper-based field effect transistor (FET), which used NCC paper as both substrate and gate dielectric layer. The FET presents a high mobility of 7 cm^2^ V^−1^s^−1^ and an on/off modulation ratio of above 10^5^. The obtained performance is comparable to those results on ordinary paper. The NCC film FET also possessed good stability. Without encapsulation or a passivation layer, the device works well under ambient conditions after one week.

### 3.5. Organic light-emitting diodes

**Figure 8.**
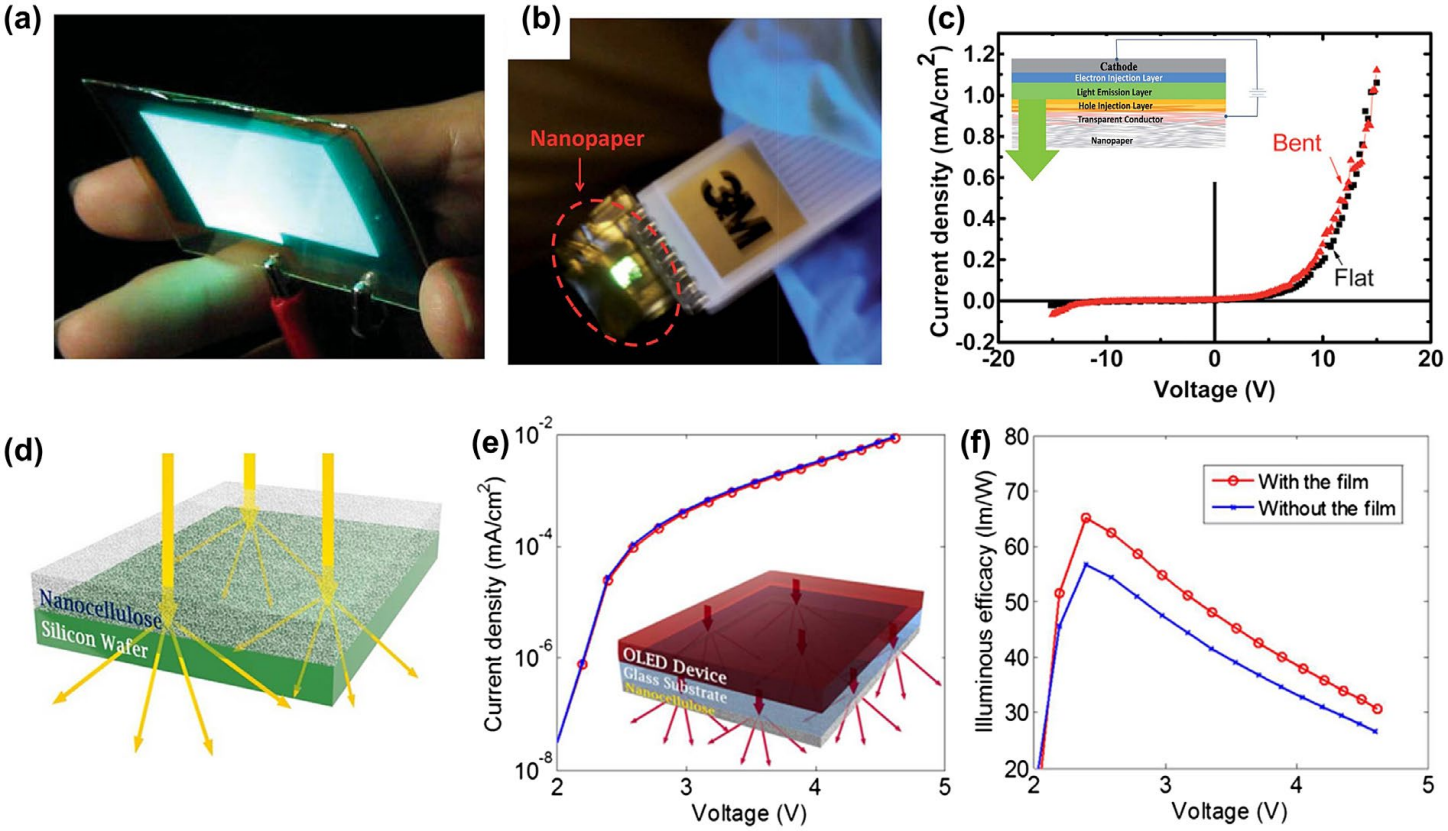
(a) Luminescence of an OLED fabricated on transparent BNC nanocomposite paper [50]. Reproduced with the permission of [50]. Copyright 2008 Wiley-VCH. (b) Image of an OLED deposited on pure nanopaper in operation. (c) I-V curve of flexible OLED based on transparent nanopaper in flat and bent states, respectively. The inset is the schematic of a nanopaper OLED device [71]. Reproduced with the permission of [71]. Copyright 2013 The Royal Society of Chemistry. (d) Schematic of the light trapping effect of nanocellulose translucent film attached on the Si wafer. (e) Current density and voltage curves with and without nanocellulose film. (f) The effect of film on illuminous efficacy-voltage curves [73]. Reproduced with the permission of [73]. Copyright 2015 American Chemical Society.

OLEDs are promising for commercialization in displays and lighting with their light-weight, thin and energy efficient characteristics. Usually, OLEDs are fabricated on glass or plastic film substrates like PET and polyethylene naphthalate. However, the glass substrate cannot be used in the roll-to-roll fabricating process, and the large coefficient of thermal expansion of plastic films results in thermal instability, which is unfavorable for OLEDs. As a recyclable and sustainable material, nanopaper is an attractive substrate for OLEDs due to its excellent thermal stability, light weight, flexibility, high optical transmittance and compatibility with roll-to-roll manufacturing. Nogi et al. [23,50] reported an OLED in 2008 using a BNC resin composite film as substrate, as shown in Figure 8(a). Unfortunately, the device is not flexible. Later, they produced a flexible film with impregnated polyurethane or acrylic resin to achieve a flexible OLED [50]. However, these devices are based on composite paper. More recently, flexible OLEDs based on 100% pure nanocellulose have been reported. Zhu et al. [71] demonstrated highly flexible OLEDs on 100% CNF nanopaper, as shown in Figure 8(b). The structure of the device is shown in Figure 8(c). The transparent conductive CNT layer was prepared by bar-coating with 10 nm thermally evaporated MoO_3_ and 30 nm poly(3,4-ethylenedioxythiophene): poly(styrenesulfonate) (PEDOT: PSS) by spin-coating at the hole injection layer. The light-emitting layer was prepared by drop-coating a green polyfluorene solution. The J-V curves at flat and bent conditions are shown in Figure 8(c). There are only small changes before and after bending, exhibiting good flexibility and mechanical stability. However, the device efficiency is low and the lighting uniformity is poor, which should be improved by reducing the resistance of the transparent conductor layer and enhancing the fabrication processes. Purandare et al. [72] fabricated a high brightness phosphorescent OLED on transparent and flexible cellulose paper by using phosphorescent Ir(PPy)_3_ as the emitting material. The current and luminous emission efficiencies can reach as high as 47 cd A^−1^ and 20 lm W^−1^, respectively. A maximum brightness of 10000 cd m^−2^ can be achieved. Wu et al. [73] prepared a highly translucent and light-diffusive nanocellulose film. The film possesses a high optical transmittance and haze, which is suitable as a translucent diffuser to improve light extraction from OLEDs (Figure 8(d)). Figure 8(e) shows the current density-voltage curves of the device with and without attaching a cellulose film with a thickness of ~ 40 μm on the glass substrate; the largest efficacy improvement can reach about 15% (Figure 8(f)). These excellent results show that low-cost and environmentally friendly transparent nanopaper is a promising substrate for transparent and flexible OLED devices.

### 3.6. Other applications

**Figure 9.**
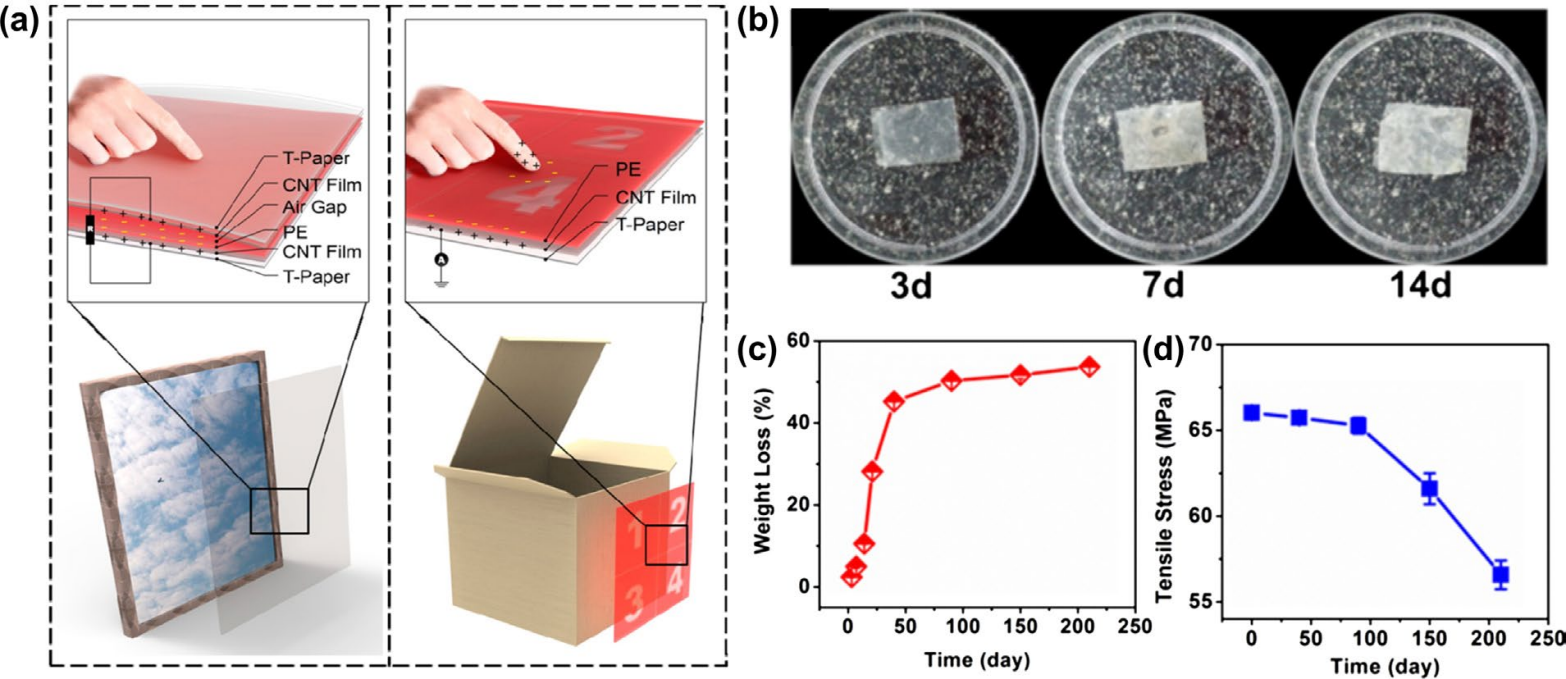
(a) Schematic illustrating the transparent nanopaper-based actuator for art anti-theft and mapping anti-forgery applications [75]. Reproduced with the permission of [75]. Copyright 2015 American Chemical Society. (b) Digital images of the degradation process for transparent nanopaper. (c) The weight loss of the nanogenerator device in soil. (d) The tensile strength change of PLA in soil [76]. Reproduced with the permission of [76]. Copyright 2016 American Chemical Society.

Paper-based generators are very attractive for energy harvesting due to their flexibility, light weight, and capability with being conformably adhered to arbitrary surfaces. There have been reports using regular paper to prepare generators which can be attached to the pages of a book collecting energy when the pages are turned [74]. However, these generator devices are not transparent. Recently, Zhong et al. [75] designed and demonstrated a self-powered and human-interactive transparent system by using transparent nanopaper made from nanocellulose. The highly transparent generator is sensitive to changes in pressure and can be integrated with valuable artworks in museums for antitheft applications (Figure 9(a)). Due to its high transmittance, the appearance of the artwork will not be affected. Zhong et al. [75] also demonstrated a possible application in smart mapping anti-forgery systems (Figure 9(a)), which have the ability to show information coding and mapping on items such as birth certificates and smart packaging. Later, they designed a biodegradable and low-cost nanogenerator by using transparent cellulose nanopaper and a polylactic acid (PLA) electret [76]. Due to the high transparency and desirable output performance, this device can be used as a self-powered smart packaging system. Furthermore, the nanogenerator can be simply buried in natural soil without further treatment (Figure 9(b)). As shown in Figure 9(c), the nanopaper will be almost completely degraded after 40 days. The mechanical strength of PLA is also decreased after 90 days (Figure 9(d)), indicating the start of structural damage.

**Figure 10.**
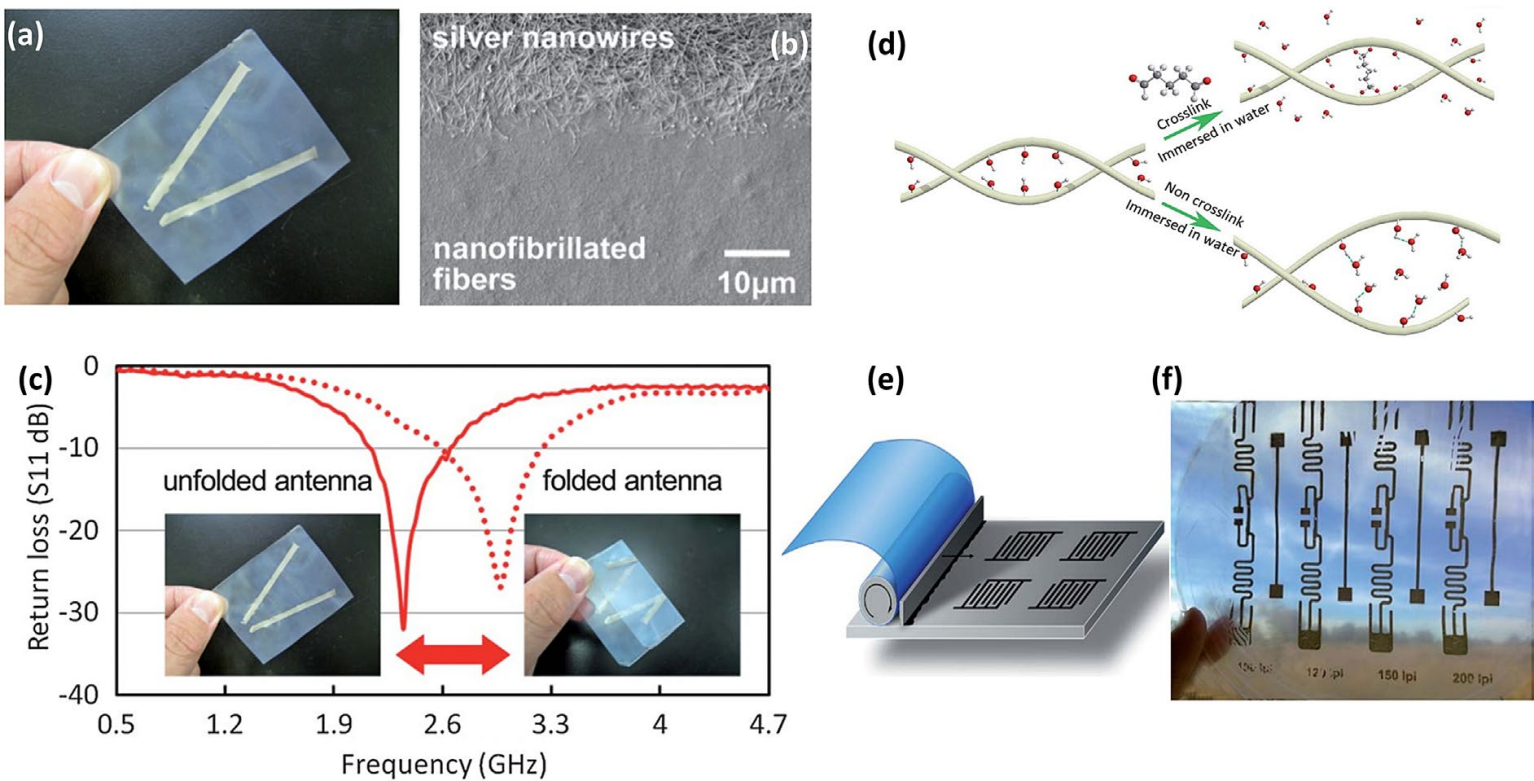
(a) The V-shaped antenna by screen-printing silver nanowire ink on transparent nanopaper. (b) Scanning electron microscopy image of the silver nanowire on nanopaper. (c) Return loss of nanopaper antennae under folded and unfolded states [77]. Reproduced with the permission of [77]. Copyright 2013 The Royal Society of Chemistry. (d) The mechanism of the shape stability improves with glutaraldehyde treatment on transparent nanopaper. (e) Schematic of the gravure printing proofer. (f) Digital photo of four RFID antennas on cross-linked nanopaper [79]. Reproduced with the permission of [79]. Copyright 2014 The Royal Society of Chemistry.

Antennae can convert electric power to radio waves and vice versa, which have been broadly used in various communication systems, such as portable phones, computers and radios. In order to meet the development of next-generation flexible and wearable electronics, the size and weight of antennae should be reduced. Due to its foldability, light weight and low roughness, nanopaper is an excellent substrate to print small and flexible antennae. Nogi et al. [77] demonstrated a V-shaped antenna by screen-printing silver nanowire ink on the smooth surface of nanopaper, as shown in Figure 10(a). The shape of the printed silver ink edge is well defined, which shows excellent printability of the nanopaper (Figure 10(b)). The antenna exhibits good sensitivity with a loss less than −26 dB. The return losses of nanopaper antennae versus frequency plot is shown in Figure 10(c). Later, to shorten the length of the antennae, they prepared a high-k nanopaper composite substrate by using NFC and Ag nanowires [78]. Compared with the antennas printed on the regular nanopaper, the silver antennae prepared on the nanopaper composite substrate can be downsized to about half of the original size. The device also exhibited the lowest return loss of around 2.60 GHz. However, the nanopaper is not stable in water, which hinders its application in large-scale printing processes, such as screen-printing and inkjet printing. To improve the shape stability of nanopaper in water, Zhu et al. [79] used hydrochloric acid (HCl) as a catalyst to cross-link the hydroxyl groups between the cellulose nanofibers (Figure 10(d)). A radio frequency identification (RFID) pattern was deposited on the dimensional stable nanopaper by gravure printing (Figure 10(e)). Figure 10(f) shows four RFID antennas printed on the transparent nanopaper. The maximum gain of the antennae was obtained at a frequency of 683 MHz and the insertion losses were about −37.9 and −38.8 dB for 100 lines per inch (lpi) and 120 lpi resolution, respectively.

Non-volatile memory is an essential component in portable and free-standing electronic components. Nagashima et al. [80] proposed an ultra-flexible resistive non-volatile memory device using Ag-decorated CNF nanopaper. The device exhibits stable non-volatile memory effects and excellent mechanical flexibility without degradation after being bent down to a radius of 350 μm. This study paves the way for the development of inexpensive, environmentally friendly and mechanically flexible memory devices for portable flexible electronics.

## 4. Conclusions and outlook

Nanocellulose is a promising material to prepare renewable and biodegradable electronics devices, and has become a hot research topic for the development of the next generation of ‘green’ flexible electronics. Compared with regular paper, the low roughness of nanopaper makes it promising for printable electronics with high printing quality. Great progress has been achieved recently. In particular: (1) various types of nanocelluloses have been developed, and different types of pure or composite transparent nanopaper with superior mechanical and optical properties have been reported; (2) various proof-of-concept devices have been demonstrated by using pure or composite transparent nanocellulose paper as a substrate with excellent flexibility, bendability and even foldability; and (3) the optical properties of transparent nanopaper have been tailored to satisfy specific electronic devices. However, there are still tremendous challenges which need be overcome before transparent nanopapers can be widely deployed in electronic devices, including the following: (1) compared with glass and plastics, the cost of pure nanopaper can be relatively high, more efficient methods should be developed to reduce the time and energy consumed to prepare the nanopaper and new processes need to be developed for high-volume extraction and high-speed fabrication of transparent nanopapers; (2) due to the hydrophilic property of nanocellulose, the shape, humidity stability and shelf life of transparent nanopaper are not satisfactory, and these problems should be resolved to reduce storage costs; and (3) printing processes should be developed for high-speed roll-to-roll coating processes on transparent nanopaper, and system integrations should also be investigated with multiple device components built on transparent nanopaper. With widespread and intensive efforts, low-cost and light-weight ‘green’ electronics fabricated on transparent nanopaper substrates will provide new technologies impacting our daily life.

## Funding

This work was financially supported by the National Research Foundation Competitive Research Programme [grant number NRF-CRP-13-2014-02] under the National Research Foundation, Prime Minister’s Office, Singapore.

## Disclosure statement

No potential conflict of interest was reported by the authors.
